# Upregulation of RasGRF1 ameliorates spatial cognitive dysfunction in mice after chronic cerebral hypoperfusion

**DOI:** 10.18632/aging.204654

**Published:** 2023-04-12

**Authors:** Li-Jie Yang, Wei Wu, Wan-Rong Jiang, Cheng-Liang Zhu, Zhao-Hui Yao

**Affiliations:** 1 Department of Geriatrics, Renmin Hospital of Wuhan University, Wuhan 430060, China; 2 Department of Clinical Laboratory, Renmin Hospital of Wuhan University, Wuhan 430060, China

**Keywords:** RasGRF1, cognition impairment, chronic cerebral hypoperfusion, synaptic plasticity, vascular cognitive impairment

## Abstract

Chronic cerebral hypoperfusion (CCH)-mediated cognitive impairment is a serious problem worldwide. However, given its complexity, the underlying mechanisms by which CCH induces cognitive dysfunction remain unclear, resulting in a lack of effective treatments. In this study, we aimed to determine whether changes in the expression of RasGRF1, an important protein associated with cognition and synaptic plasticity, underlie the associated impairments in cognition after CCH. We found that RasGRF1 levels markedly decreased following CCH. Through prediction and validation studies, we observed that miRNA-323-3p was upregulated after CCH and could bind to the 3′-untranslated region of *Rasgrf1* mRNA and regulate its expression *in vitro*. Moreover, the inhibition of miRNA-323-3p upregulated *Rasgrf1* expression in the hippocampus after CCH, which was reversed by *Rasgrf1* siRNA. This suggests that miRNA-323-3p is an important regulator of Rasgrf1. The Morris water maze and Y maze tests showed that miRNA-323-3p inhibition and *Rasgrf1* upregulation improved spatial learning and memory, and electrophysiological measurements revealed deficits in long-term potentiation after CCH that were reversed by *Rasgrf1* upregulation. Dendritic spine density and mature mushroom spine density were also improved after miRNA-323-3p inhibition and *Rasgrf1* upregulation. Furthermore, *Rasgrf1* upregulation by miRNA-323-3p inhibition improved dendritic spine density and mature mushroom spine density and ameliorated the deterioration of synapses and postsynaptic density. Overall, RasGRF1 regulation attenuated cognitive impairment, helped maintain structural and functional synaptic plasticity, and prevented synapse deterioration after CCH. These results suggest that *Rasgrf1* downregulation by miRNA-323-3p plays an important role in cognitive impairment after CCH. Thus, RasGRF1 and miRNA-323-3p may represent potential therapeutic targets for cognitive impairment after CCH.

## INTRODUCTION

Vascular cognitive impairment (VCI) is highly prevalent among persons of advanced age, and its incidence increases as the population ages [1]. As is well known, Alzheimer’s disease (AD) and vascular dementia (VaD) are the top two types of dementia in persons above age 65, based on the pathogenesis. Furthermore, between 20% and 40% patients with dementia diagnosis and no less than 40% of patients with AD diagnosis concomitantly display VCI [2]. VaD is the most severe form of VCI. Different conditions cause VCI, such as cerebral hypoperfusion, embolic occlusion, hypertension, and vasculopathy [3]. Chronic cerebral hypoperfusion (CCH) is one of the main pathophysiological causes of VCI. Dysfunctional protein expression regulation is believed to reinforce cognitive dysfunction after CCH. Various signaling pathways (ferroptosis [4], autophagy [5], and oxidative stress [6]) and cognition-related proteins (MeCP_2_ [7], HIF-1 [8], hyperphosphorylated tau [9], and BDNF [10]) have been implicated in this process. However, despite these diverse potential targets, current therapies have proven ineffective. Synaptic plasticity is fundamental for normal cognition, and its impairment underlies many signaling pathway changes that contribute to cognitive dysfunction [11]. Previous studies have shown that CCH also affects synaptic plasticity [12]. However, the mechanism involved is still largely unknown, and a critical target for treating cognitive deficit has yet to be found.

Ras guanine nucleotide releasing factor 1 (RasGRF1) is a neuron-specific guanine-nucleotide releasing factor that enhances Ras and Rac activity. RasGRF1 is widely distributed in the brain, especially in the neurons of the hippocampus and corpus striatum [13–15], and is closely related to spine formation and cognition [16, 17]. *Rasgrf1* expression promotes neurite outgrowth after neurotrophin induction [18], dendritic spine formation [19–21], axonal growth and branching [22], and long-term potentiation (LTP) [23]. *Rasgrf1* knockout mice showed significantly impaired olfactory associative memory [24], defective contextual fear conditioning memory in hippocampus-dependent learning and memory experiments [14, 25], and significantly reduced conditioned place preference [26]. Ethanol ingestion during pregnancy also impaired the memory of adult male offspring and reduced *Rasgrf1* expression [27]. Furthermore, intractable epilepsy reduced the level of brain RasGRF1 [28]. Treatment with Δ9-tetrahydrocannabinol reduced the RNA and protein levels of RasGRF1 and impaired cognition [29, 30]. Reduced RasGRF1 levels led to nuclear condensation and transcription inhibition in neurons [31]. These studies suggest that RasGRF1 homeostasis is important for normal cognition and neuropsychiatric function. However, whether RasGRF1 contributes to cognitive impairment after CCH remains to be determined.

MicroRNAs (miRNAs) are noncoding RNAs, each consisting of 19–22 nucleotides, that are generated by RNA polymerase II and III and further processed by the ribonucleases Drosha and Dicer. miRNA binds with Dicer and Argonaute proteins to form the RNA-induced silencing complex (RISC) to guide targeted mRNAs to degrade or inhibit translation [32]. miRNAs are widely distributed throughout the body, including the brain, cerebrospinal fluid, and blood, and play an important role in regulating protein expression in the central nervous system. Dysregulation of miRNAs has been implicated in neurodegenerative diseases, blood–brain barrier damage, neuroinflammation [33–35], synaptic transmission [36], LTP [37], and cognition [38]. However, whether miRNAs downregulate *Rasgrf1* after CCH remains unknown. Therefore, in this study, we investigated RasGRF1 levels after CCH to explore the underlying mechanisms of CCH-induced cognitive impairment and to determine new effective therapeutic targets.

## MATERIALS AND METHODS

### Animals

The 3-month 87 Sprague Dawley rats were purchased from Vital River Laboratories (Beijing, China). The animals can have the food and drink water freely. The housing condition has the 12 hours dark/light alternate cycles. All animal experiment ethics are approved by the Animal Ethics Committee of Renmin Hospital of Wuhan University and comply with the animal experimental guidelines of NIH.

**Figure 1:**
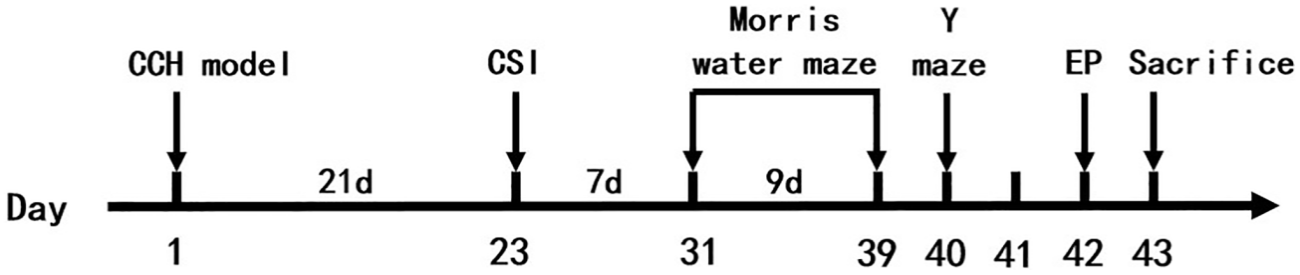
**Experimental procedures and timeline.** The rats were treated by 2VO and sham surgery. 21 days (3 weeks) later, the cerebral stereotaxic injection was done. 7 days (1 week) later, the Morris water maze and Y maze were done. 1 day later, electrophysiological experiments were done on rats. Next day, the rats were executed and then be tested by Western blot, RT-PCR, immunohistochemistry, Golgi staining and transmission electron microscopy. Abbreviations: CCH: chronic cerebral hypoperfusion; CSI: cerebral stereotaxic injection; EP: electrophysiology.

The animals were randomly divided into 6 groups: sham control group (Con, *n* = 12), bilateral common carotid arteries occlusion group (2VO, *n* = 15), 2VO with miRNA-323-3p inhibitor (antagomir) intra-cerebroventricular injection (icv) group (2VO+miRNA-323-3p antagomir, *n* = 15), 2VO with miRNA-323-3p antagomir negative control icv group (2VO+ miRNA-323-3p antagomir NC, *n* = 15), 2VO with miRNA-323-3p antagomir and RasGRF1 siRNA icv group (2VO+miRNA-323-3p antagomir + RasGRF1 siRNA, *n* = 15) and 2VO with miRNA-323-3p antagomir and RasGRF1 siRNA scramble icv group (2VO+miRNA-323-3p antagomir + RasGRF1 scramble siRNA, *n* = 15). All experimental procedures were arranged in Figure 1.

### Chronic cerebral hypoperfusion model

After the rats were anesthetized with 0.6% Pelltobarbitalum Natricum ((w/v) × 100%), the bilateral common carotid arteries were separated softly with the vagus nerve from sheath. And then the double 4-silk thread went through under the bilateral common carotid arteries. The bilateral threads in both sides were ligated in one hour time interval. After occlusion, the arteries were put back to primary places in sheathes. The incisions were sutured and sterilized, and the rats were placed on the heating pad for anesthetization recovery to return house cages. Chronic cerebral hypoperfusion models were considered to be successfully made when cerebral blood flow in the brains of the operated rats was detected by the ultrasound Doppler system and cerebral blood flow reduced to less than 70% of normal values. In this study, the success rate of CCH model making was 90%.

### Chemicals and reagents

The rabbit polyclone antibody against RasGRF1 (Cat.No:12958-1-AP) was from Proteintech group (Wuhan, China) for Western blot. The rabbit polyclone antibody against RasGRF1 (Cat.No: GB11856) was from Servicebio company (Wuhan, China) for immunohistochemistry. The mouse monoclonal antibody against β-actin was purchased from Cell signaling Technology, Inc. (Danvers, MA, USA). The labeled HRP secondary antibodies against rabbit and mouse IgG were from Biosharp Life Sciences (Hefei, Anhui, China). Biotin-labeling goat antibody against rabbit IgG and HRP-labeling streptavidin were from Zymed Technologies (San Diego, CA, USA). The diaminobenzidine (DAB) detection kit was from ZSbio.ltd. (Beijing, China). Mimics (miRNA-323-3p, miRNA-375-3p, and miRNA-448-5p), miRNA-323-3p antagomir and negative control were from RiboBio company (Guangzhou, China). RasGRF1 siRNA and scramble siRNA were designed and synthesized by Shanghai Genechem Co., Ltd. (Shanghai, China).

### Morris water maze

It has been shown that 4-week CCH can induce cognitive dysfunction in rats [39]. In the present study, after all rats were subjected to CCH for 30 days, the Morris water maze was employed to test the spatial learning and memory. The detailed procedure was as following [40]. The rats were accommodated with maze environment one day before the maze training. Then the rats will be trained during 7 days. The maze was divided into 4 quadrants in clockwise orientation. On each day, the rats were placed slowly into every maze quadrant along the maze wall to swim, at which point they searched for the platform. When the rats reached the platform in 60 s, they would learn to remember the platform location with referring the around signs for 10 s. If the animals did not find the platform in 60 s, they would be guided to the platform for remember the platform location. During the 7-day training, the latency time to find the platform in 60 s would be recorded to evaluate the rats’ spatial learning ability. After finishing the training, the platform was removed. After resting for 1 day, they were again placed into the maze to record the latency time, the time crossing the platform quadrant, and the staying time in the platform quadrant, for evaluating the short-term spatial memory.

### Y maze

The Y maze consist three arms with 45 cm length, 15 cm width and 25 cm height, as well as one joint platform. The Y maze had two-phase tests. For one phase, one arm was shut down and the rats were placed into maze to walk freely for 5 min in other two arms. Then after the rat rested for 10 min in house cages, the closed arms were opened and the rats was again placed in the maze to freely explore all three arms for 5 min. The number entering all arms (n) would be counted. The three continuously different arms entries would be as one correct alternate. The number of total correct alternate was designated as c. The maximum possible correct alternate number was equal to n-2. The correct alternate percent was calculated as (c/(n-2)) × 100%. Total correct alternate number and the correct alternate percent were used to evaluate the rats’ short-term spatial learning and memory ability [41].

### Cerebral stereotaxic injection

Cerebral stereotaxic injection was done 3 weeks after the 2VO occlusion. The rats were anesthetized with 0.6% Pelltobarbitalum NatricumChloral ((w/v) × 100%) and the median incision was made along the skull. The bregma was exposed with 3% H_2_O_2_. The rats’ head placed on the stereotaxic apparatus and the skull plane was kept horizontal. The lateral ventricle coordinate was as: anteroposterior (AP) 1.2 mm, mediolateral (ML) ± 1.6 mm, DV (dorsoventral) 4.4 mm. The small holes were made by the cranial drill according to the coordinates. The microsyringe lifted down 4.4 mm from the skull surface through the holes. The siRNAs, miRNA mimics and inhibitor were injected using the microsyringe at 50 nl/min for 20 min. After finishing the injection, the microsyringe remained in the brain for 5 min to prevent the drugs from back flowing along the needle holes. After the holes were stuffed up with vaseline, the incision was closed and the antibiotic was applied on the skin to avoid infection [42].

### Cell culture and transfection

The rat pheochromocytoma cell line cells (PC12) were cultured in Dulbecco’s modified Eagle’s medium (DMEM) supplemented with 5% fetal calf serum (FCS) and 10% horse serum under humidified atmosphere of 5% CO_2_ in 37°C constant temperature incubator. PC12 cells were transfected with the pGL3-Basic plasmid containing the wild type or mutated 3′UTR fragment of RasGRF1 and pRL-TK plasmid in transfection agent Lipofectamine 2000 (TermoFisher Scientifc, USA). After the 6-h transfection, the culture medium was change with the fresh DMEM medium. Fishing 24-h the transfection, the PC12 cells were used for dual luciferase reporter assay and collected to extract total proteins for Western blot.

### Dual luciferase reporter assay

The 3′UTR of gene was cloned into the downstream of luciferase gene in reporter vector pGL3-Basic with firefly luciferase gene. pRL-TK was as the inner control report vector with Renilla luciferase gene. After these vectors were together in PC12 cells, the medium was incubated with miRNA mimics. The florescence with 560 nm wave length was detected to evaluate the luciferase expression level in pGL3 (over 480 nm wave length florescence from Renilla luciferase). The strength of the relative intensity of florescence was negatively correlated with the binding and translational regulation between miRNAs and the 3′UTR of target genes [43, 44].

### Western blot

After finishing the behavior tests, the rats were anesthetized to death with 0.6% Pelltobarbitalum Natricum ((w/v) × 100%). The brains were gotten rapidly form skull and hippocampi were separated into liquid nitrogen for storage. Then the hippocampi tissue was homogenized in homogenization buffer containing the proteinase inhibitor with frozen grinder. The homogenate was mixed with 4 times loading buffer and boiled for 10 min for protein denaturation. After centrifuging for 10 min with 12000 g, the supernatant was gotten for the assay of protein concentration by BCA protein assay kit and mixed with mixture of β-mercaptoethanol and bromophenol blue to re-boiled for 5 min denaturation. The 30 μg total protein was loaded into SDS-gel for 1 h electrophoresis with 100 mA current. And the proteins in the gel were transmembraned into PVDF membrane with 270 mA current. The proteins on the PVDF were developed with primary antibody RasGRF1 for overnight under 4°C. After washing the superfluous antibody, the membranes were developed with HRP-labeled secondary antibody for 1 hour under the room temperature. After washing, the membrane was developed with ECL kit and detected under the Bio-Rad membrane scanner for relative analysis of proteins content [45].

### RNA extraction, library preparation, RNA sequencing and differentially expressed RNAs analysis

Hipocampi tissue of the rats’ brain was obtained and stored at −80°C until use. Small RNA was extracted and RNA purities were evaluated using the NanoDrop 2000 spectrophotometer (Thermo Fisher Scientific, Waltham, MA, USA). RNA integrities were assessed using the Agilent 2100 Bioanalyzer (Agilent Technologies, Santa Clara, CA, USA). Then the libraries were constructed using TruSeq Stranded Total RNA with Ribo-Zero Gold (Illumina, Cat.No. RS-122-2301) and were sequenced on an Illumina HiSeq X Ten platform. After removing low quality reads, clean sequencing reads were mapped to the human genome (GRCh38) using HISAT2 [46]. For miRNAs, FPKM was calculated using Cufflinks [47]. Differential expression analysis was performed using the DESeq (2012) R package [48]. *p* value < 0.05 was as the threshold for significantly differential expression.

### Quantitative reverse transcription polymerase chain reaction (RT-PCR)

After extraction of total RNAs in 50 mg hippocampal tissue with TRIzol (Invitrogen, Singapore), complementary DNA (cDNA) was synthesized by a First-strand cDNA Synthesis Kit (Thermo Fisher Scientific, Waltham, MA, USA). Along with cDNA primers, RT-PCR was carried out with the SYBR GREEN Mix reaction system (Thermo Fisher Scientific, Waltham, MA, USA) [49]. The reaction process was as following: 2 min pre-incubation at 95°C; an amplification step (45 cycles of 95°C for 20 s, 57.5°C and 72°C for 30 s); an elongation step (72°C for 10 min). The absence of primer dimers was verified by fitting melting curve. SnRNA U6 was the endogenous control for miRNA and Gapdh was the endogenous control for RasGRF1. The primers for miRNAs, RasGRF1 and mutated 3′UTR were listed (Table 1). The “ΔΔ Ct method” was used to assay the relative RNA expression [50].

**Table 1:** The primers used in present study.

**Target**	**Primer**	**Length**
rno-miR-21-3p	5′-GACAGCCCATCGACTGCTGTTG-3′	22bp
rno-miR-137-3p	5′-CTACGCGTATTCTTAAGCAATAA-3′	23bp
rno-miR-375-3p	5′-TCACGCGAGCCGAACGAACAAA-3′	22bp
rno-miR-195-3p	5′-TGGAGCAGCACAGCCAATATTGG-3′	23bp
rno-miR-323-3p	5′-AGAGGTCGACCGTGTAATGTG-3′	21bp
rno-miR-203a-3p	5′-CTAGTGGTCCTAAACATTTCAC-3′	22bp
rno-miR448-5p	5′-TGGCAGCACTATGCAGGATGTT-3′	22bp
rno-miR-423-5p	5′-TTTTGTCTCGCTCTCTGCCCCTCT-3′	24bp
rno-miR-137-3p	5′-CTACGCGTATTCTTAAGCAATAA-3′	23bp
rno-miR-132-3p	5′-CGTCCCTTGGCTGTTGTCTGTTT-3′	23bp
rno-miR-152-3p	5′-CCTTGTTCTGTCTGCTCTGT-3′	20bp
rno-miR-207	5′-TTTGGGTGGTGTGCCTHHTGTTG-3′	23bp
rno-miR-484	5′-TTCGGGTGGGGTCTGTGCTGT-3′	21bp
Rasgrf1 mutated 3′UTR	5′-ACTACAAGATTACATGGC-3′	18bp
Rasgrf1 F	5′-CATCTACCAGGAGTTCGTCC-3′	20bp
Rasgrf1 R	5′-ATTTGGCATAGTCCAGGC-3′	18bp
Rasgrf1 siRNA	5′-GGAGGAGATTGATATGACC-3′	19bp
SnRNA U6 F	5′-GCTTCGGCAGCACATATAC-3′	19bp
SnRNA U6 R	5′-CTTCACGAATTTGCGTGT-3′	18bp

### Immunohistochemistry

The rats were perfused with 4% paraformaldehyde through cardiac puncture after anesthetization. And then the brains were gotten from the skulls for overnight post fixation. The brains were dehydrated with 20%, 30%, and 30% sucrose solution. The dehydrated brains were rapidly frozen in the liquid nitrogen for sectioning with freezing microtome. The 25 μm slices were gotten and develop with primary antibody for overnight under 4°C. Then the slices were developed with the biotin-labeled secondary antibody for 1 h under 37°C. The slices were developed with HRP-labeling streptavidin and stained with DAB kit. After gradient dehydration, the sliced were mounted on the slide with neutral balsam and observed under the microscope. The positive neurons were counted for analysis [49].

### Electrophysiology

The rats were anesthetized with 0.6% pentobarbital sodium ((w/v) × 100%). Synaptic plasticity in the entorhinal cortex (EC) -CA1 pathway was investigated. CA1 coordinate was as: AP 3.2 mm, ML ± 2.4 mm, DV 3.4 mm. EC perforant path (PP) coordinate was as: AP 8.3 mm, ML ± 3.9 mm, DV 4.4 mm. The stimulating electrode was implanted in PP and the dual recording electrodes were implanted in CA1. The maximum evoked field excitable postsynaptic potential (fEPSP) was recorded and the 60% intensity of the stimulating current was employed to give the high frequency stimulation (HFS). The fEPSP was recorded with 2kHz sampling speed. Baseline fEPSP was recorded for 10 min. After the HFS (12 trains of 15 stimuli (200 Hz) with 5-s intervals) was imposed, fEPSP was recorded for 60 min. LTP was analyzed with the ratio of the post-HFS fEPSP slope over baseline slope [49]. The average fEPSP and population spike slope over the baseline slope were calculated. Data were processed with Igor Pro 6.1 (WaveMetrics, Lake Oswego, OR, USA) software.

### Golgi staining

The rats were anesthetized with 0.6% Pelltobarbitalum Natricum ((w/v) × 100%) and perfused with 4% formaldehyde solution. The brains were taken out and sectioned into 3~5 mm piece of tissue. The tissues were immersed in Golgi staining solution (consist of 4% formaldehyde, 5% potassium bichromate and 5% chloral hydrate) for 14 days and silver-stained for 7 days. And the stained tissues were cut into 80 μm slices. The slices were washed enough in water to avoid the superfluous impurities. Three to five dendrites in the each of 30 cells per animal were observed. Dendritic spines and mushroom spines in a particular dendritic branch were counted in different the focus. Spine density is designated as spines number in 10 μm. The cumulative distribution of spines density was calculated and visualized by R package.

### Transmission electron microscopy

After anesthetization with 0.6% Pelltobarbitalum Natricum ((w/v) × 100%), the rats were perfused with cold fixation solution (phosphate buffer containing 2% paraformaldehyde and 1.25% glutaraldehyde). The hippocampi were separated for re-fixation in cold 2.5% glutaraldehyde PB overnight and. post-fixation in 1% osmium tetroxide. After dehydration in graded ethanol and acetone solutions, the tissues were embedded in epoxy resin and sectioned into 70 nm ultrathin slices for negative stain with uranyl acetate and lead citrate. The hippocampus slices were observed under transmission electron microscope (HT7700, Hitachi, Japan). The synapses in the certain interest area were counted to evaluate the synapse density and the postsynaptic density (PSD) was measured in length, area and intensity to analyze the synapse microstructure [51]. The cumulative distribution of synapse length and area was calculated and visualized by R package.

### Statistics

Data were described as means ± SEM and statistically analyzed with SPSS 16.0 statistical software (SPSS Inc., Chicago, IL, USA). The repeated measures analysis of variance was employed to assay the statistical differences of the mean learning latency time in different groups. For other studies data, difference comparisons in two groups were calculated by a two-tailed Student’s *t* test. Cumulative distribution differences for total spines density and synapse length and area between different groups were evaluated with the Kolmogorov-Smirnov test by R software [52]. *P* < 0.05 was indicated as statistically significant difference.

### Data availability

All the data used to support the findings of this study are available from the corresponding author upon request.

## RESULTS

### RasGRF1 levels in the hippocampus markedly decreased after CCH

**Figure 2:**
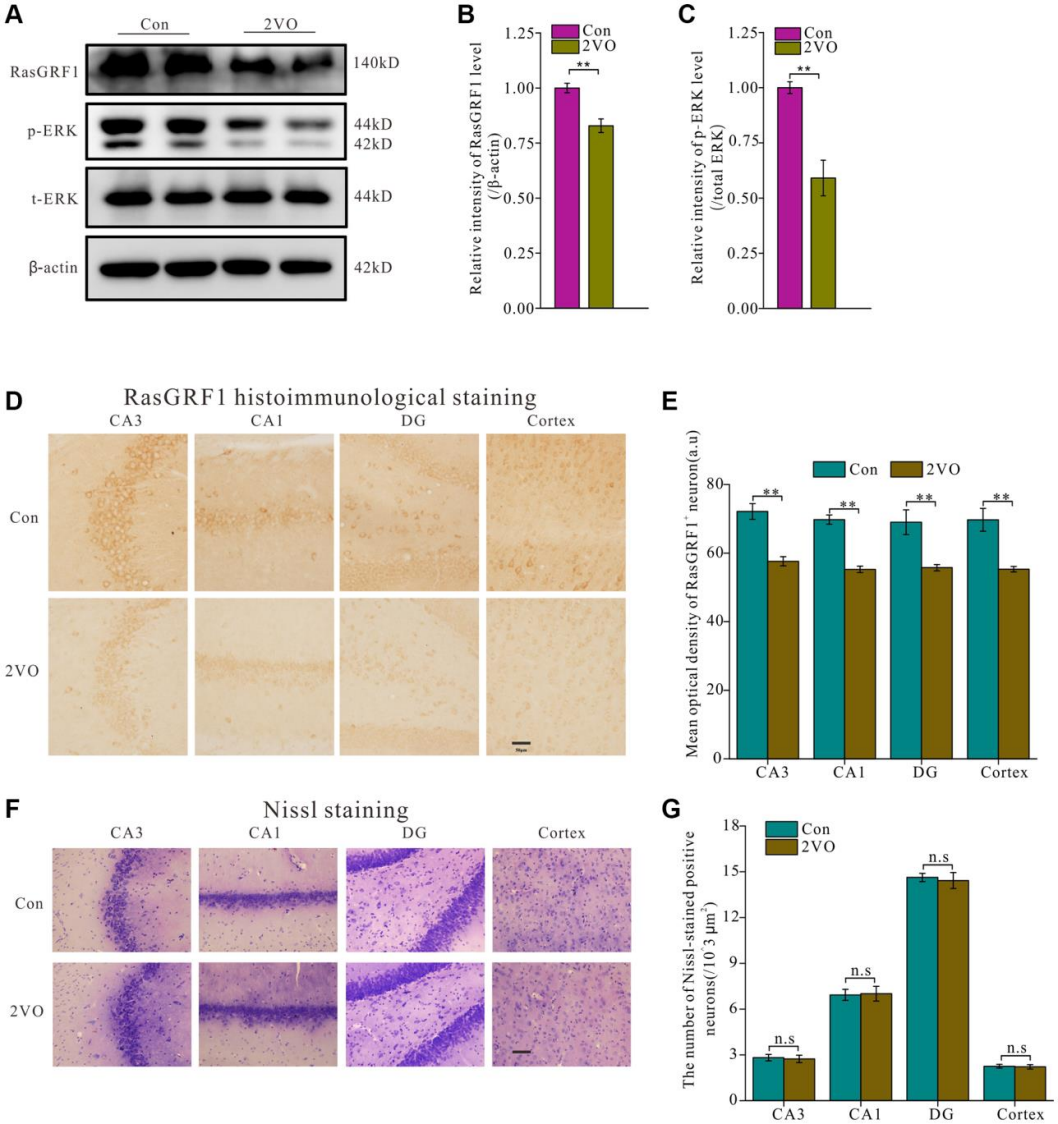
**RasGRF1 expression in hippocampus dramatically decreased after chronic cerebral hypoperfusion.** (**A**) The RasGRF1 expression, the total and phosphorylated ERK expression were assayed by Western blot. β-actin was as inner control for samples loading normalization; (Con (*n* = 3), 2VO (*n* = 3)). (**B**) The RasGRF1 relative expression level was calculated and analyzed statistically. (**C**) The p-ERK relative expression level was calculated and analyzed statistically. Relative intensity of p-ERK level is calculated by p-ERK/total ERK. Total ERK was as inner control for samples loading normalization. (**D**) RasGRF1 distribution in brain was observed with immunohistochemical staining; (Con (*n* = 1), 2VO (*n* = 1)). (**E**) In hippocampal subregions and cortex, the RasGRF1-positive cells were counted and analyzed. (**F**) The neurons number was observed with Nissl staining; (Con (*n* = 1), 2VO (*n* = 1)). (**G**) The Nissl-stained positive cells were counted and analyzed. Compared with Control, ^**^p < 0.01. Scale bar = 50 μm. All of the experiments were repeated three times.

To investigate whether RasGRF1 levels changed after CCH, hippocampal homogenates were assayed using Western blot analysis (Figure 2A). RasGRF1 level in the 2VO group was significantly lower than that in the control group (*p* < 0.01) (Figure 2B). As ERK is the downstream effector of RasGRF1, Western blot analysis showed that the phosphorylated ERK (p-ERK) level in the 2VO group was also apparently lower than that in the control group (*p* < 0.01) (Figure 2C). Immunohistochemical staining showed that the RasGRF1 levels in the 2VO group decreased significantly compared to that in the control sham group in the hippocampal CA1, CA3, and dentate gyrus regions and in the cortex (*p* < 0.01) (Figure 2D and 2E). The loss of neurons caused by chronic cerebral ischemia may contribute to the decreased expression of RasGRF1. Nissl staining was performed to further explore the mechanism by which RasGRF1 expression decreased and revealed that the number of neurons was comparable between the 2VO and control sham groups (*p* > 0.05) (Figure 2F and 2G). It means that RasGRF1 expression decrease may not be caused by neuron loss, but by a decrease in its own level.

### miRNA-323-3p binds 3′-untranslated regions to regulate RasGRF1 expression

**Figure 3:**
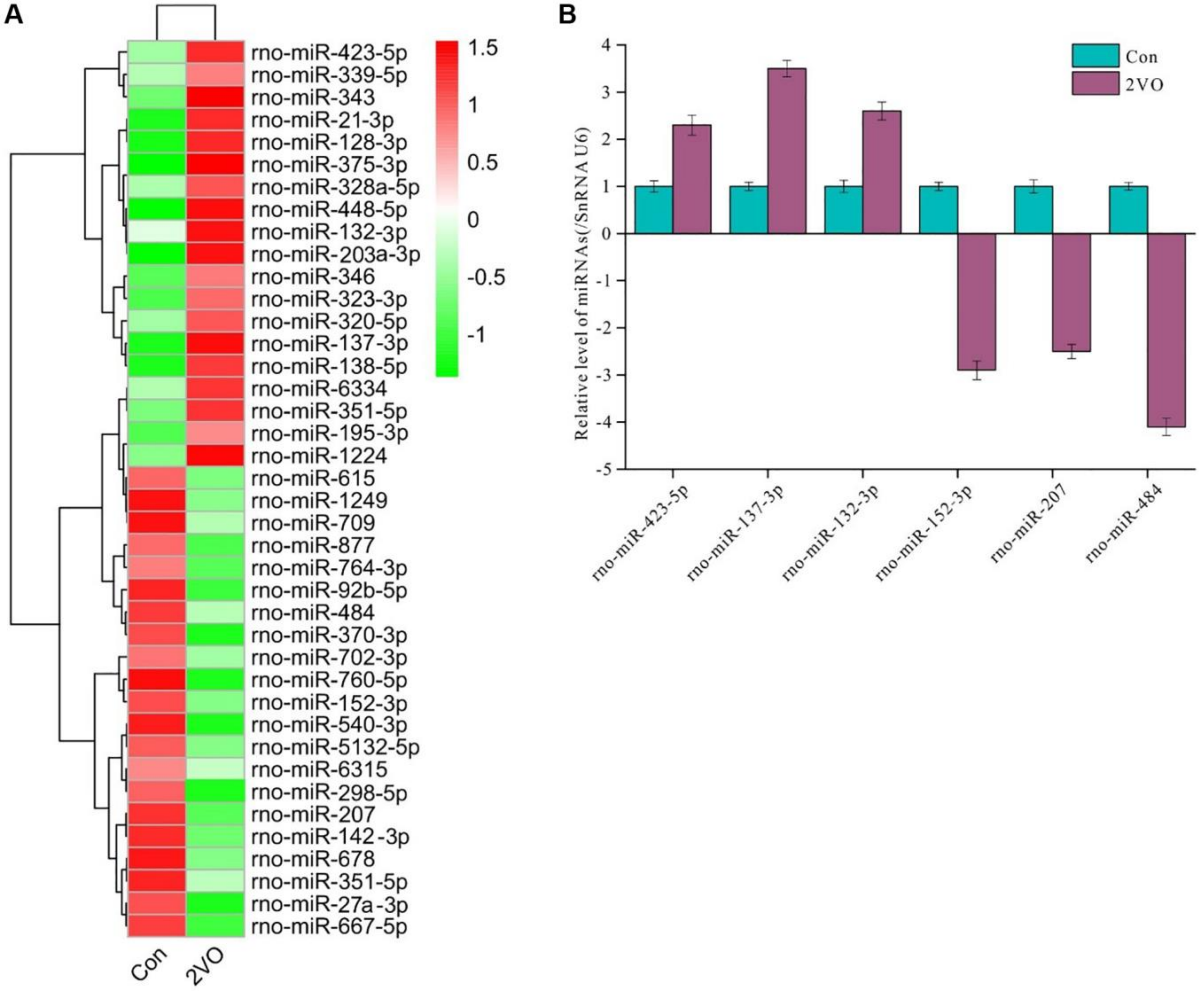
**Differentially expressed miRNAs profile in hippocampus after chronic cerebral hypoperfusion.** The hippocampal miRNAs were extracted and sequenced. (**A**) The differentially expressed miRNAs profile was displayed. (**B**) The selected differentially expressed miRNAs were validated by RT-PCR. All of the experiments were repeated three times. The experiments were repeated three times.

To explore the regulatory function of *Rasgrf1*, small RNAs in the hippocampus were extracted following behavioral test administration. Then, cDNA libraries were constructed by reverse transcription and sequenced. After processing, 40 differentially expressed miRNAs were found (Figure 3A). Of these, rno-miRNA-423-5p, rno-miRNA-137-3p, rno-miRNA-132-3p, rno-miRNA-152-3p, rno-miRNA-207, and rno-miRNA-484 were validated by RT-PCR. The RT-PCR data were consistent with the sequencing data (Figure 3B).

**Figure 4:**
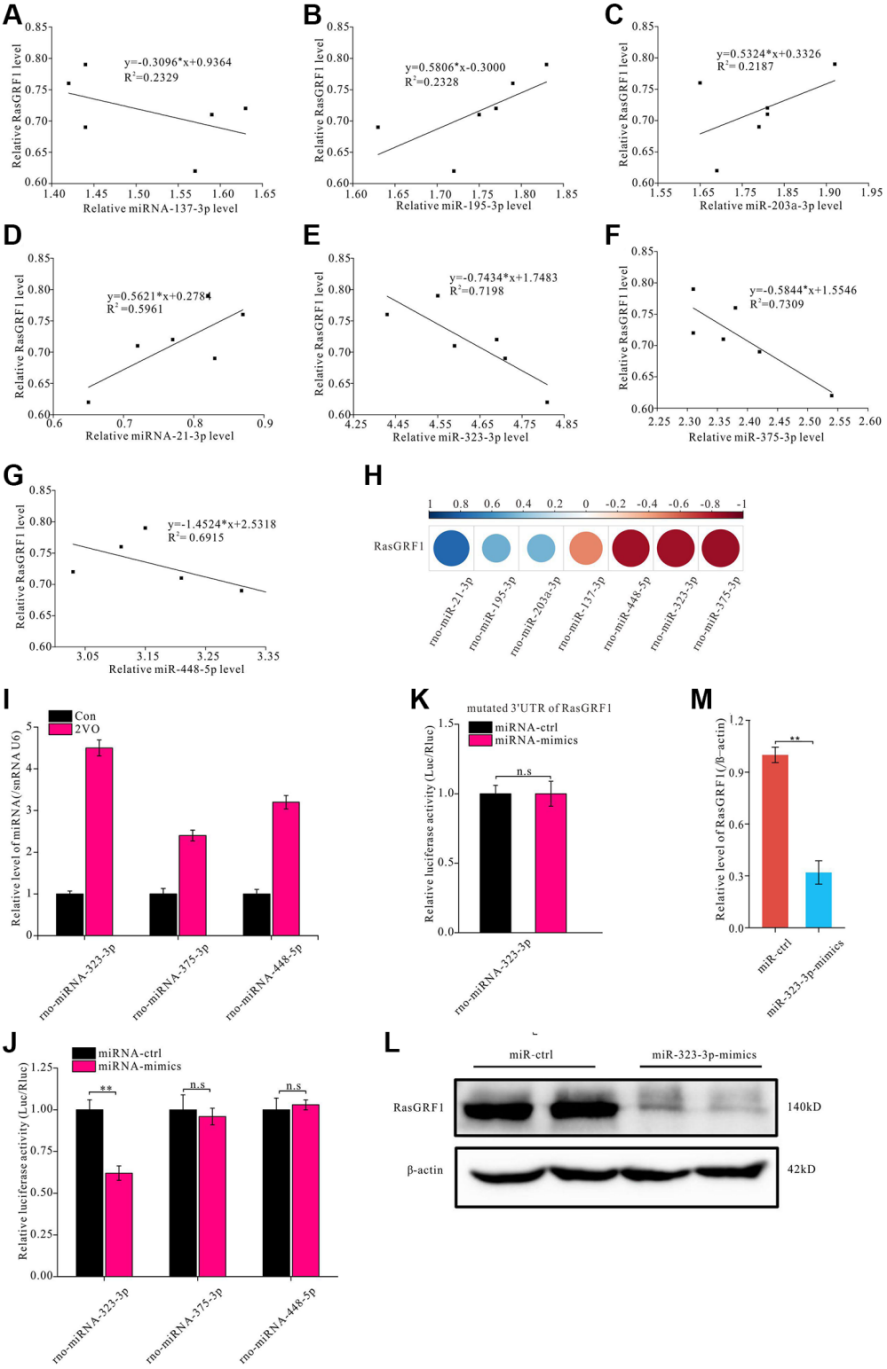
**miR-323-3p could regulate the expression of RasGRF1 through binding to the 3′UTR.** The total RNAs were extracted, and RasGRF1 and the predicted bind miRNAs levels were measured by RT-PCR. (**A**–**G**) The correlation between miRNAs (rno-miR-137-3p, rno-miR-195-3p, rno-miR-203a-3p, rno-miR-21-3p, rno-miR-323-3p, rno-miR-375-3p, rno-miR-448-5p) and RasGRF1 levels were analyzed. (**H**) The correlation degree was displayed with heatmap. (**I**) rno-miR-323-3p, rno-miR-375-3p and rno-miR-448-5p level in hippocampus after CCH were showed. (**J**) Dual luciferase reporter assayed the binding and expression regulation of targeted RasGRF1 mRNAs 3’UTR by miRNA mimics and negative control of miRNA-323-3p, miRNA-375-3p and miRNA-448-5p. (**K**) The binding and regulation between mutated 3’UTR of RasGRF1 mRNA and miRNA-323-3p was assayed by dual luciferase reporter. (**L**) RasGRF1 level after rno-miR-323-3p mimic treatment was detected by Western blot. β-actin was as inner control for samples loading normalization. (**M**) The RasGRF1 relative expression level was calculated and analyzed statistically. Compared with miRNA-Control, ^**^p < 0.01. All of the experiments were repeated three times.

miRNA is an important regulator of protein expression [53, 54]. To further investigate the mechanisms underlying RasGRF1 reduction after CCH, miRNA predictive tools (TargetScan, miRDB, and miRBase) were employed to predict miRNA binding to the 3′-UTR of *Rasgrf1* mRNA. miRNAs with the highest *Rasgrf1*-binding scores (rno-miR-137-3p, rno-miR-195-3p, rno-miR-203a-3p, rno-miR-21-3p, rno-miR-323-3p, rno-miR-375-3p, and rno-miR-448-5p) were selected for validation through RT-PCR. The correlation analysis showed that rno-miR-137-3p, rno-miR-323-3p, rno-miR-375-3p, and rno-miR-448-5p levels were negatively correlated with *Rasgrf1* expression, whereas rno-miR-195-3p, rno-miR-203a-3p, and rno-miR-21-3p levels were positively correlated (Figure 4A–4G). The correlation coefficients are shown in Figure 4H. Because the correlation coefficient between miRNA-137-3p and *Rasgrf1* mRNA was small, miRNA-323-3p, miRNA-375-3p, and miRNA-448-5p were selected for further binding analysis (Figure 4I).

A dual luciferase reporter assay was performed in PC12 cells to verify the interaction. The 3′-UTR of *Rasgrf1* was cloned downstream of the luciferase gene in a reporter vector (pGL3-Basic). p RL-TK was used as the internal control. Following transfection with the two reporter constructs, the cells were incubated with miRNA-323-3p, miRNA-375-3p, and miRNA-448-5p mimics. Relative fluorescence at 560 nm was detected to evaluate the luciferase expression level of pGL3; this showed that miRNA-323-3p could reduce the relative fluorescence activity associated with the *Rasgrf1* 3′-UTR (*p* < 0.01) (Figure 4J), but miRNA-375-3p and miRNA-448-5p could not. To further investigate the binding between miRNA-323-3p and the *Rasgrf1* 3′-UTR, the 3′-UTR was mutated and cloned into another reporter plasmid for transfection. The miRNA-323-3p mimics could not reduce the relative luciferase activity of the mutated *Rasgrf1* 3′-UTR (*p* > 0.05) (Figure 4K). RasGRF1 levels were also detected in PC12 cells incubated with miRNA-323-3p mimics using Western blotting (Figure 4L). The blot analysis showed that treatment with miRNA-323-3p mimics markedly decreased the RasGRF1 level (Figure 4M).

### miR-323-3p inhibition may increase RasGRF1 levels after CCH

**Figure 5:**
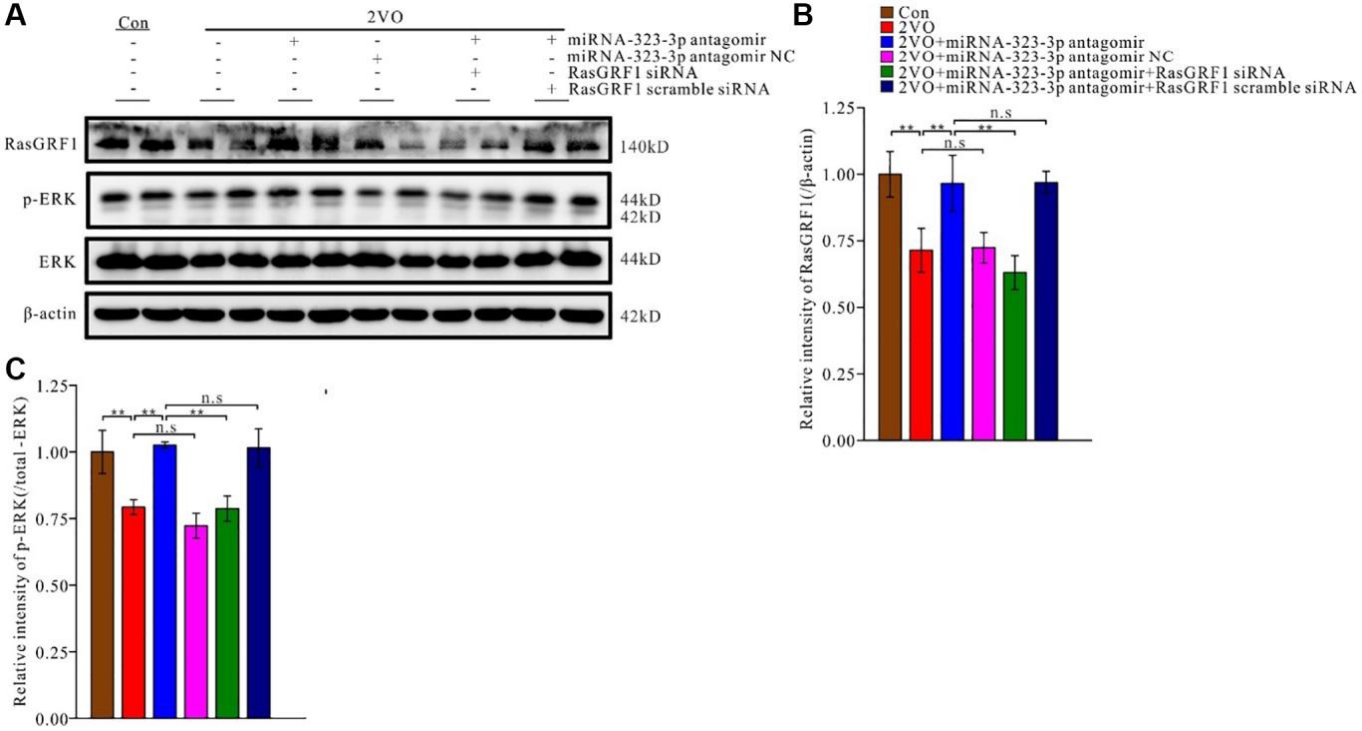
**The inhibition of miR-323-3p could up-regulate the expression of RasGRF1 in hippocampus after CCH.** (**A**) The RasGRF1 expression, the total and phosphorylated ERK expression were assayed by Western blot. β-actin was as inner control for samples loading normalization. (Con (*n* = 3), 2VO (*n* = 3), 2VO+miRNA-323-3p antagomir (*n* = 3), 2VO+ miRNA-323-3p antagomir NC (*n* = 3), 2VO+miRNA-323-3p antagomir + RasGRF1 siRNA (*n* = 3), 2VO+miRNA-323-3p antagomir + RasGRF1 scramble siRNA (*n* = 3)). (**B**) The relative expression level of RasGRF1 was calculated and analyzed statistically. (**C**) The p-ERK relative expression level was calculated and analyzed statistically. Relative intensity of p-ERK level is calculated by p-ERK/total ERK. ^**^p < 0.01. The experiments were repeated three times.

To investigate whether miR-323-3p inhibition could prevent RasGRF1 reduction after CCH, a miR-323-3p inhibitor (antagomir) was administered to rats through icv injection. RasGRF1 levels were then detected using Western blot analysis (Figure 5A). Antagomir increased RasGRF1 levels to a greater extent than the 2VO controls (*p* < 0.01) but not the antagomir negative control (*p* > 0.01). Moreover, *Rasgrf1* siRNA prevented the antagomir-induced RasGRF1 increase (*p* < 0.01), whereas the scrambled siRNA did not (*p* > 0.05) (Figure 5B).

As a downstream effector of RasGRF1, ERK participates in regulating synaptic plasticity and cognitive function. RasGRF1 can regulate ERK activation [21, 55, 56] and spine density [20, 21]. To explore the downstream regulatory pathway of RasGRF1, the levels of ERK and p-ERK in rat hippocampus were analyzed using Western blotting (Figure 5A). Treatment with antagomir, but not its negative control (*p* > 0.05), increased p-ERK levels to a greater extent than 2VO surgery alone (*p* < 0.01). *Rasgrf1* siRNA prevented the antagomir-induced p-ERK increase (*p* < 0.01), whereas the scrambled siRNA did not (*p* > 0.05) (Figure 5C).

### *Rasgrf1* upregulation by inhibition of miR-323-3p could improve learning and memory impairment after CCH

**Figure 6:**
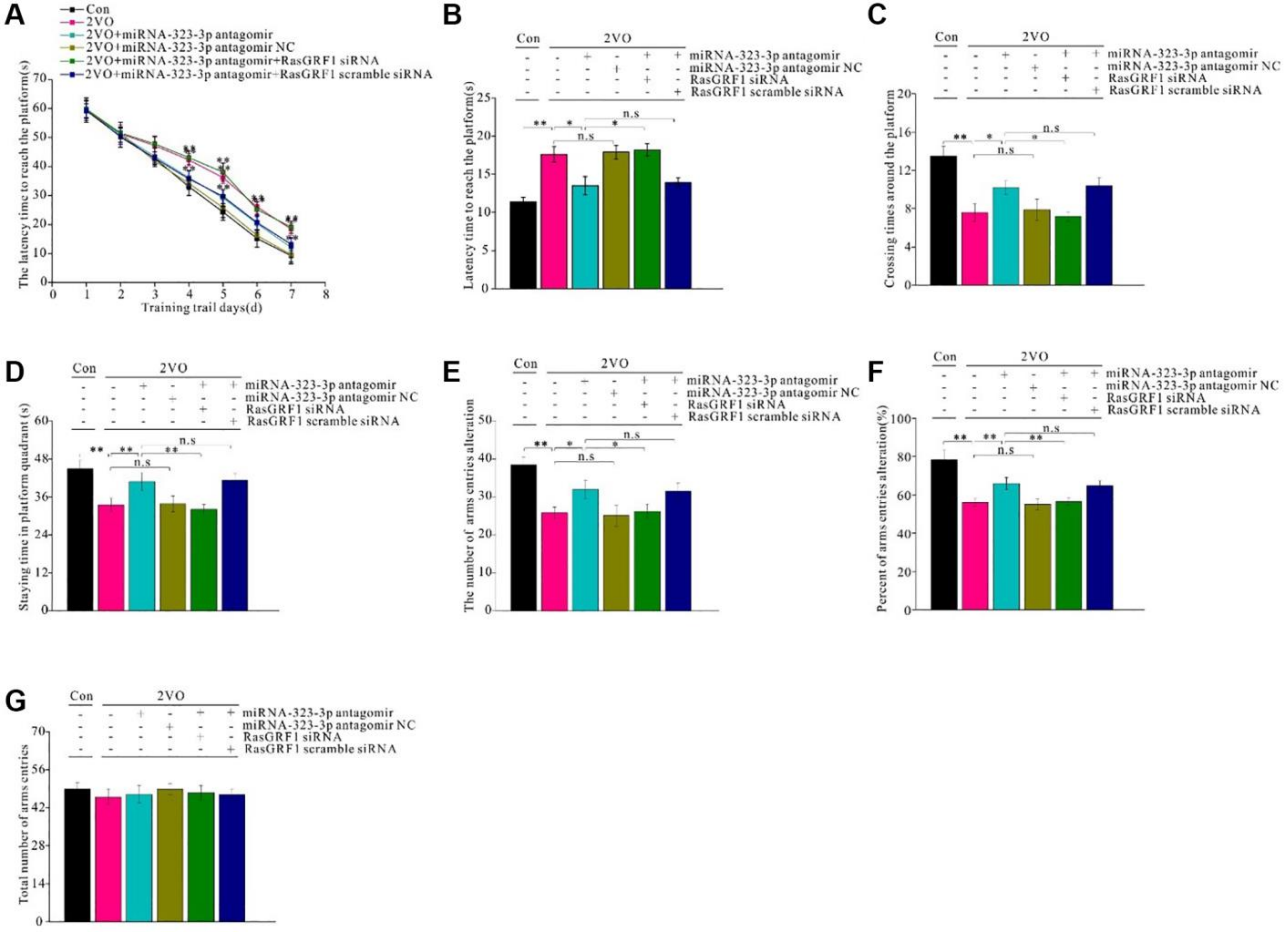
***Rasgrf1* upregulation by the inhibition of miR-323-3p could improve the spatial cognitive.** dysfunction after CCH. MWM was employed to detect the spatial cognitive abilities. (**A**) The latency to reach the platform during the trail training was recorded and analyzed. (**B**) After 1 day-rest, the latency to reach the platform. (**C**) The crossing times around the platform, and (**D**) staying time in platform quadrant were recorded and analyzed. The Y maze was employed to further test the cognitive abilities. (**E**) The number of arms entries alteration, (**F**) percent of arms entries alteration, and (**G**) total arms entries times were recorded and analyzed. Con: sham group, *n* = 12; 2VO: bilateral common arteries occlusion group, *n* = 15; 2VO+miR-323-3p antagomir: bilateral common arteries occlusion group with miR-323-3p antagomir intra-cerebroventricular injection (ICV) injection, *n* = 15; 2VO+miR-323-3p NC: 2VO group with ICV injection of miR-323-3p antagomir negative control, *n* = 15; 2VO+miR-323-3p antagomir+RasGRF1 siRNA: 2VO group with ICV injection of miR-323-3p antagomir and RasGRF1 siRNA, *n* = 15; 2VO+miR-323-3p antagomir + RasGRF1 scramble: 2VO group with ICV injection of miR-323-3p antagomir and RasGRF1 siRNA scramble, *n* = 15. ^*^p < 0.05, ^**^p < 0.01. All of the experiments were repeated three times.

To investigate whether *Rasgrf1* upregulation could improve spatial learning and ameliorate memory dysfunction after CCH, the Morris water maze was employed. No difference in swimming speed of the rats in the different groups was noted. From day 4 to 7, the 2VO group took longer time to reach the platform than did the miR-323-3p antagomir group during training trials (*p* < 0.01; Figure 6A). The negative control and 2VO groups had comparable latency times (*p* < 0.01; Figure 6A). The improved latency time in the miR-323-3p antagomir group was inhibited by *Rasgrf1* siRNA (*p* < 0.01) but not by scrambled siRNA (*p* > 0.05; Figure 6A). After 1 day of rest, the short-term memory test showed that the 2VO group had a longer latency time to reach the platform than the sham group (*p* < 0.01; Figure 6B), while the miR-323-3p antagomir group had a shorter latency time than the 2VO group (*p* < 0.01; Figure 6B). There was no significant difference in latency time between the negative control and 2VO groups (*p* < 0.01; Figure 6B). The increased latency time in the miR-323-3p antagomir group was inhibited by *Rasgrf1* siRNA (*p* < 0.01; Figure 6B) but not scrambled siRNA (*p* > 0.05; Figure 6B). The miR-323-3p antagomir group had improved crossing times around the platform and time spent in platform areas after CCH, and *Rasgrf1* siRNA administration could prevent these improvements (*p* < 0.01; Figure 6C and 6D).

To further evaluate spatial learning and memory, the Y maze was employed. miR-323-3p antagomir treatment increased the number and percent of arm entries after CCH, but *Rasgrf1* siRNA treatment prevented this increase (*p* < 0.01; Figure 6E and 6F. There was no difference in the total number of arm entries between all groups (*p* < 0.05; Figure 6G).

### *Rasgrf1* upregulation by inhibition of miR-323-3p improved synaptic plasticity impairment after CCH

**Figure 7:**
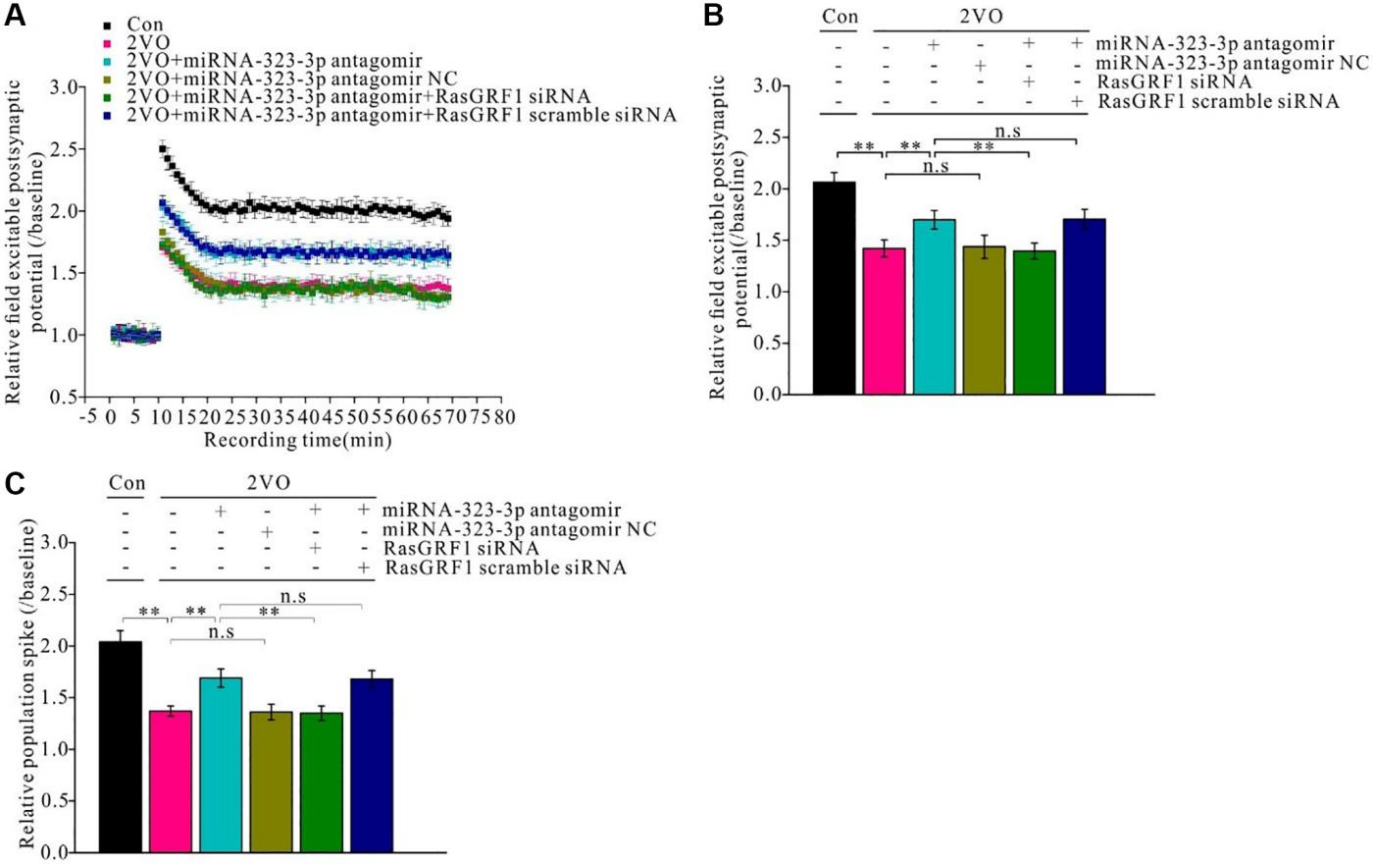
***Rasgrf1* upregulation by the inhibition of miR-323-3p could improve the long-term potential impairment after CCH.** (**A**) Field excitable postsynaptic potential was recorded and analyzed pre- and post- high frequency stimulation (pre-HFS recording as the baseline). (Con (*n* = 3), 2VO (*n* = 3), 2VO+miRNA-323-3p antagomir (*n* = 3), 2VO+ miRNA-323-3p antagomir NC (*n* = 3), 2VO+miRNA-323-3p antagomir+RasGRF1 siRNA (*n* = 3), 2VO+miRNA-323-3p antagomir + RasGRF1 scramble siRNA (*n* = 3)). (**B**) Relative field excitable postsynaptic potential and (**C**) population spike was analyzed over the baseline. ^**^p < 0.01. The experiments were repeated three times.

To investigate the mechanism of *Rasgrf1* upregulation during spatial cognitive dysfunction after CCH, electrophysiological techniques were employed to measure synaptic functional plasticity. Pre- and post-high-frequency stimulation trains were applied to hippocampal slices *in vivo* and field excitatory postsynaptic potentials (fEPSPs) were recorded and analyzed (Figure 7A). The relative fEPSPs and population spikes over baselines were measured and calculated. The fEPSP reduction after CCH improved with administration of miR-323-3p antagomir (*p* < 0.01) but not its negative control (*p* > 0.01). Further, *Rasgrf1* siRNA could prevent this improvement in the antagomir group (*p* < 0.01), whereas the scrambled siRNA could not (*p* > 0.05; Figure 7B and 7C).

### *Rasgrf1* upregulation by inhibition of miR-323-3p improved dendritic spine deterioration after CCH

**Figure 8:**
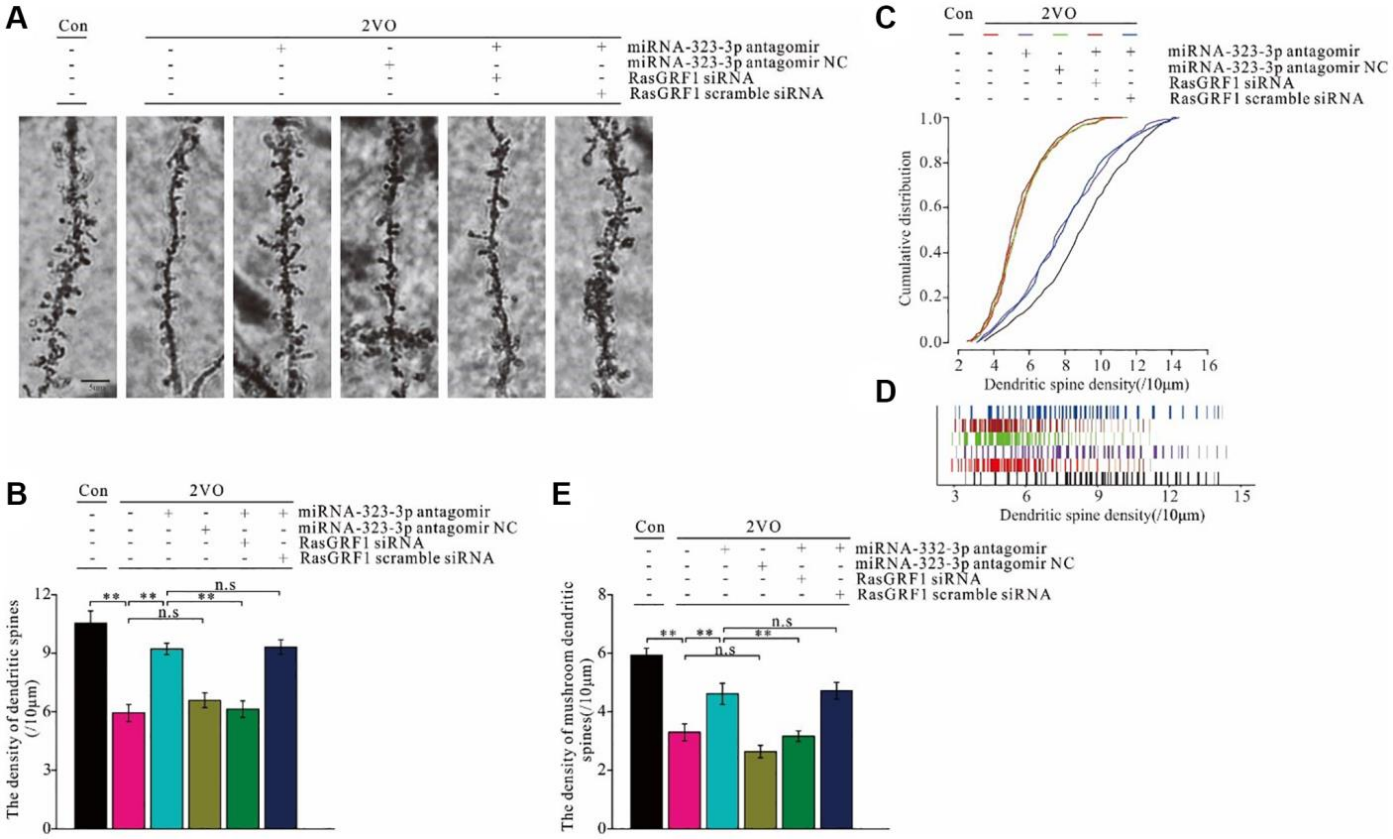
***Rasgrf1* upregulation by the inhibition of miR-323-3p could ameliorate the neural structural plasticity impairment after CCH.** (**A**) The dendritic spines were observed with Golgi staining. (**B**) The cumulative distribution of dendritic spines density was calculated; (Con (*n* = 1), 2VO (*n* = 1), 2VO+miRNA-323-3p antagomir (*n* = 1), 2VO+ miRNA-323-3p antagomir NC (*n* = 1), 2VO+miRNA-323-3p antagomir + RasGRF1 siRNA (*n* = 1), 2VO+miRNA-323-3p antagomir + RasGRF1 scramble siRNA (*n* = 1)). (**C**) The total dendritic spines density. (**D**) The cumulative distribution of dendritic spines density. (**E**) The mushroom dendritic spines density was counted and calculated. Scale bar = 5 μm. ^**^p < 0.01. All of the experiments were repeated three times.

Synaptic structural plasticity is the foundation of neuronal functional plasticity, which is an important underlying trait for learning and memory formation. Since RasGRF1 contributes to dendritic spine formation [17, 19], Golgi staining was employed to observe dendritic spines and investigate alterations in synaptic structural plasticity caused by miRNA-323-3p inhibition (Figure 8A). The total number of dendritic spines was counted, and spine density was analyzed. Dendritic spine density after CCH increased with administration of miRNA-323-3p antagomir (*p* < 0.01) but not the negative control (*p* > 0.05). This improvement of total dendritic spine density was prevented by *Rasgrf1* siRNA treatment in the antagomir group (*p* < 0.01) but not by scrambled siRNA (*p* > 0.05; Figure 8B). The decrease of the cumulative distribution of dense dendritic spines after CCH was improved by miRNA-323-3p antagomir treatment (*p* < 0.01) but not the negative control (*p* > 0.05). This improvement in the antagomir group was prevented by *Rasgrf1* siRNA (*p* < 0.01) but not scrambled siRNA (*p* > 0.05; Figure 8C and 8D). miRNA-323-3p and *Rasgrf1* siRNA had similar effects on mushroom spine density and the total number of dendritic spines (Figure 8E).

### *Rasgrf1* upregulation by inhibition of miR-323-3p improved synapse deterioration after CCH

**Figure 9:**
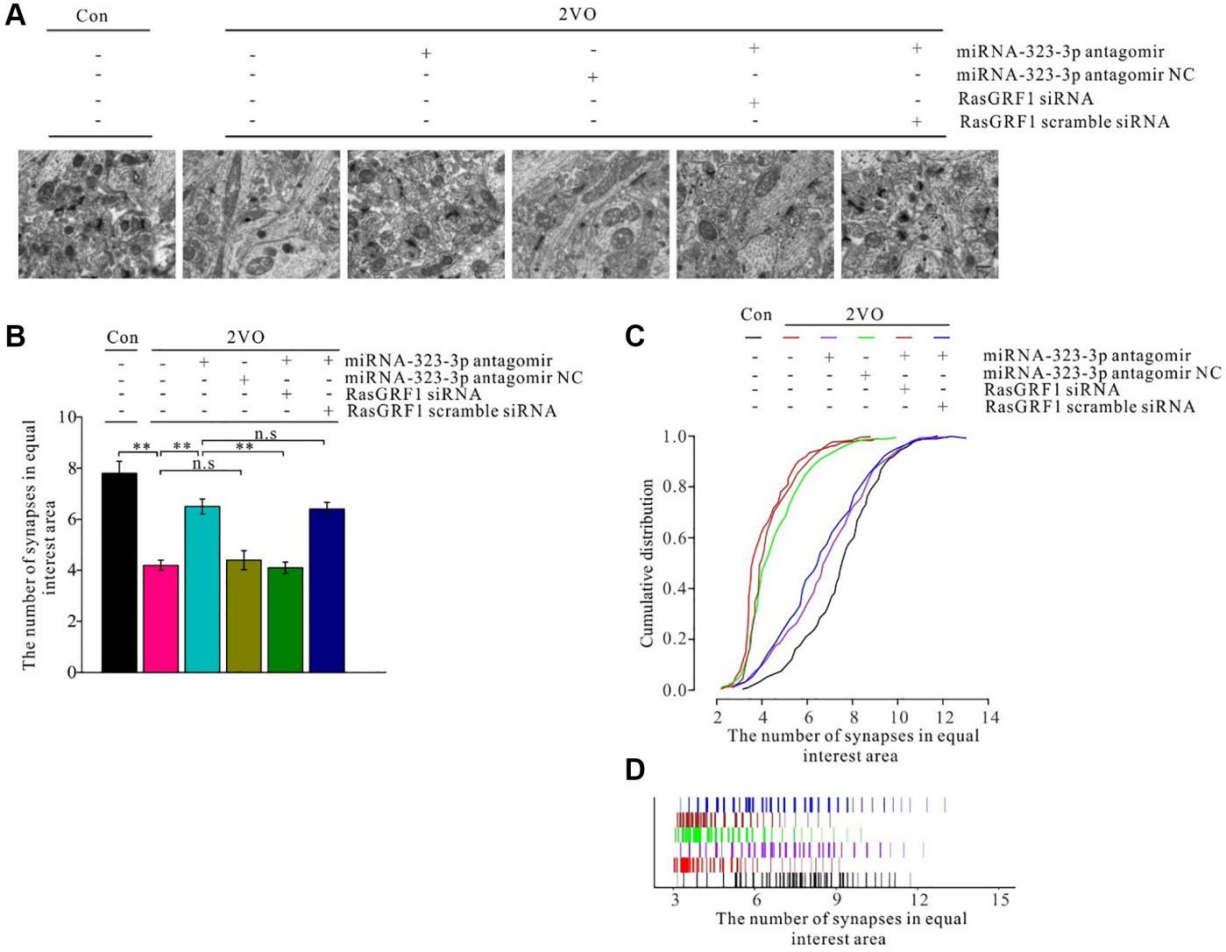
***Rasgrf1* upregulation by the inhibition of miR-323-3p could improve the synapse density decrease after CCH.** (**A**) The synapses were observed with uranyl acetate negative staining under transmission electron microscope; (Con (*n* = 3), 2VO (*n* = 3), 2VO+miRNA-323-3p antagomir (*n* = 3), 2VO+miRNA-323-3p antagomir NC (*n* = 3), 2VO+miRNA-323-3p antagomir + RasGRF1 siRNA (*n* = 3), 2VO+miRNA-323-3p antagomir + RasGRF1 scramble siRNA (*n* = 3)). (**B**) Synapses densities were calculated. (**C** and **D**) The cumulative and specific distribution of synapses density was calculated. Scale bar = 200 nm. ^**^p < 0.01. All of the experiments were repeated three times.

A previous study showed synapse degeneration after CCH [57, 58]. To investigate whether *Rasgrf1* upregulation could ameliorate synapse structural degeneration after CCH, the synapses were observed under TEM with negative staining (Figure 9A). Synaptic density after CCH increased on treatment with miRNA-323-3p antagomir (*p* < 0.01) but not the negative control (*p* > 0.05). The improvement of synaptic density in the antagomir group was prevented by *Rasgrf1* siRNA (*p* < 0.01) but not scrambled siRNA (*p* > 0.05; Figure 9B). Synaptic density in the 2VO group was much lower than that in the sham group (*p* < 0.01), while 2VO with the miRNA-323-3p antagomir group had more areas of high density than did the 2VO group (*p* < 0.01). However, the 2VO with the negative control group did not have more areas of high density than did the 2VO group (*p* > 0.05). The increase in synaptic density in the antagomir group was prevented by *Rasgrf1* siRNA (*p* < 0.01) but not by scrambled siRNA (*p* > 0.05; Figure 9C and 9D).

**Figure 10:**
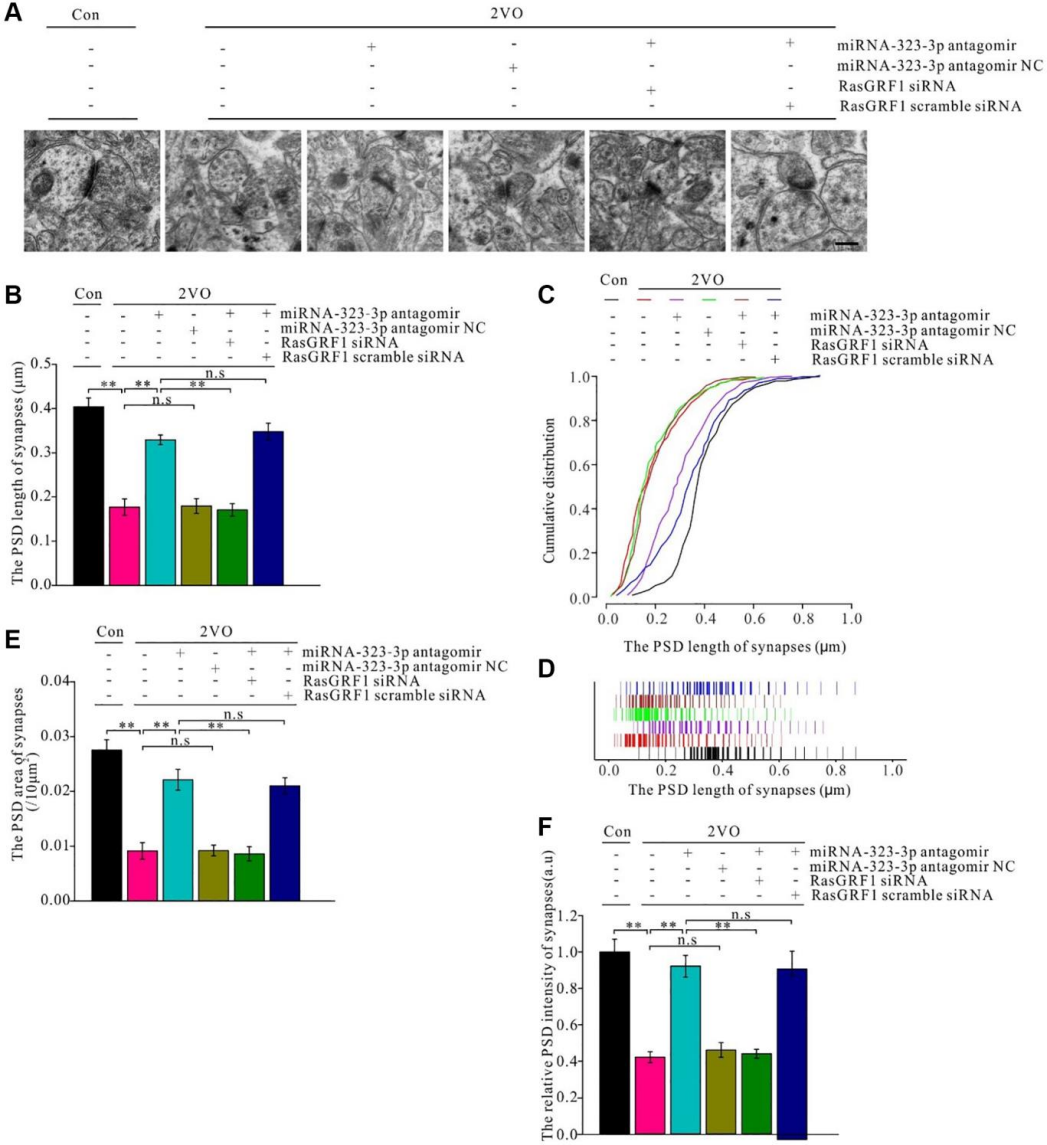
***Rasgrf1* upregulation by the inhibition of miR-323-3p could improve the synapse structure deterioration after CCH.** (**A**) The synapses were observed with uranyl acetate negative staining under transmission electron microscope and the synapses’ structure was observed; (Con (*n* = 3), 2VO (*n* = 3), 2VO+miRNA-323-3p antagomir (*n* = 3), 2VO+ miRNA-323-3p antagomir NC (*n* = 3), 2VO+miRNA-323-3p antagomir + RasGRF1 siRNA (*n* = 3), 2VO+miRNA-323-3p antagomir + RasGRF1 scramble siRNA (*n* = 3)). (**B** and **C**) The cumulative and specific distribution of synaptic PSD length. (**D**) PSD length of synapses. (**E**) Synapse area and (**F**) the relative PSD intensity of synapses were measured and calculated. Scale bar = 200 nm. ^**^p < 0.01. All of the experiments were repeated three times.

The postsynaptic density (PSD) is important for synapse excitation. After CCH, synaptic length increased with administration of miRNA-323-3p antagomir (*p* < 0.01; Figure 10A) but not the negative control (*p* > 0.05). This increase in the antagomir group was prevented by *Rasgrf1* siRNA (*p* < 0.01) but not scrambled siRNA (*p* > 0.05; Figure 10B). Moreover, the 2VO group had a higher distribution of short synapses than the sham group (*p* < 0.01); however, miRNA-323-3p antagomir increased the distribution of long synapses in the 2VO group (*p* < 0.01) compared with that in the negative control (*p* > 0.05). Meanwhile, synapses in the antagomir group had a higher distribution of short synapses when treated with *Rasgrf1* siRNA (*p* < 0.01) but not scrambled siRNA (*p* > 0.05; Figure 10C and 10D). After CCH, the PSD area and intensity increased with miRNA-323-3p antagomir but not the negative control (*p* > 0.05). The improvement of the PSD length, area, and intensity in the antagomir group was inhibited by *Rasgrf1* siRNA but not scrambled siRNA (*p* < 0.01; Figure 10E and 10F).

## DISCUSSION

This study showed that RasGRF1 levels decreased after CCH. RNA sequencing showed 40 miRNAs as being differentially expressed after CCH. Of these, only miRNA-323-3p had the capacity to bind to the 3′-UTR of *Rasgrf1* and regulate its expression. Further validation *in vivo* and *in vitro* showed that miRNA-323-3p could indeed inhibit *Rasgrf1* expression. In addition, miRNA-323-3p inhibition by antagomir could upregulate *Rasgrf1* in the hippocampus after CCH. This RasGRF1 regulation could improve cognitive impairment, functional and structural synaptic plasticity, and synapse deterioration after CCH.

miRNA is an important factor that regulates the expression of mRNA through binding the 3′-UTR of mRNA. Previous studies showed that RasGRF1 could be regulated by miR-137 to inhibit astrocytoma [59]. However, in the present study, several different algorithms predicted that RasGRF1 has many potential miRNA-regulated sites. The correlation coefficients showed that the correlation coefficient of miRNA-323-3p, miRNA-375-3p, and miRNA-448-5p were large. Our dual luciferase reporter assay further validated that miRNA-323-3p could bind to *Rasgrf1* and decrease the overall RasGRF1 level. The inhibition of miRNA-323-3p also increased *Rasgrf1* expression and cognitive dysfunction after CCH. Although miRNA-375-3p, and miRNA-448-5p were also predicted to have high binding scores with RasGRF1, some factors may influence the binding, such as spatial conformation and RNA modifications. These findings suggest that miRNA-323-3p likely underlies RasGRF1 reduction after CCH, providing a novel mechanism for RasGRF1 regulation and a potential therapeutic target for cognitive dysfunction after CCH.

A previous study showed that RasGRF1 had a differentially methylated region (DMR) at the promoter region and that DMR hypermethylation silenced RasGRF1 gene expression [60]. In a kainate-induced epileptogenesis model, *Rasgrf1* was downregulated following DMR hypermethylation, but this was blocked by RG108, a non-nucleoside DNA methyltransferase (DNMT) inhibitor. In a study evaluating the CCH brain, the global DNMT level showed dynamic changes and increased to 30% at 90 days [61]. Thus, it was postulated that RasGRF1 might be regulated through DMR hypermethylation; however, whether the downregulation of *Rasgrf1* is indeed triggered by DMR hypermethylation after CCH remains unclear. In the future, we plan to investigate this further. Furthermore, CDK5 phosphorylates RasGRF1 at Ser731 to decrease the level of steady-state RasGRF1 and degrade it as part of a calpain-dependent mechanism. One study demonstrated that when p35/CDK5 was overexpressed or active, RasGRF1 levels decreased [31]. Additionally, p35/CDK5 signaling was activated after CCH [62, 63]. These data suggest that RasGRF1 may be phosphorylated by CDK5 and subsequently degraded and downregulated after CCH. However, this needs to be verified in future studies.

RasGRF1 is closely associated with cognitive function. Mutant mice with inactivated RasGRF1 had impaired hippocampus-dependent learning and memory [14], while *Rasgrf1* knockout mice had reduced neuronal ERK activity and displayed memory deficits [64]. RasGRF1 elimination also caused significant gene expression changes in processes related to neural signal transduction [65]. Moreover, a single-nucleotide variant in RasGRF1 (rs8027411) was associated with the change of human memory function [16]. The present study showed that *Rasgrf1* upregulation from miRNA-323-5p inhibition could improve spatial learning and memory dysfunction, suggesting that *Rasgrf1* downregulation plays a critical role in spatial cognition dysfunction after CCH and that miRNA-323-5p may be an effective therapeutic target for cognitive dysfunction after CCH.

Synaptic plasticity is known to have a fundamental role in the maintenance of normal cognitive function. RasGRF1 participates in regulating synaptic plasticity. RasGRF1 is activated by calmodulin and cytosolic free calcium and interacts directly with the C-terminus of NR2B [66]. This interaction may activate ERK by phosphorylation through p38 MAPK signaling to promote LTP in neurons [56, 67] and plays a key role in regulating neuronal synaptic plasticity. As such, blocking RasGRF1 with an inhibitor could suppress LTP induction by inhibiting ERK phosphorylation [68]. The present study showed that the reduction of the phosphorylated active form of ERK was prevented following *Rasgrf1* upregulation by miRNA-323-3p inhibition in the CCH model. Moreover, *Rasgrf1* upregulation by miRNA-323-3p inhibition could improve the deficit in LTP after CCH. These data suggest that *Rasgrf1* downregulation potentially reduces ERK activation and contributes to LTP impairment after CCH. Moreover, a previous study showed that protein level of NR2B decreased after CCH [69], perhaps exacerbating the deterioration of ERK activation by weakening the interaction of RasGRF1 with NR2B. Furthermore, AMPA glutamate receptors have also been shown to activate RasGRF1 to regulate Ras/ERK signaling [70]. Muscarinic receptors activate G protein-coupled receptors and increase the phosphorylation of RasGRF1 at Ser916/898, which is required for RasGRF1 activation [71]. In CCH animal models, the levels of both AMPA receptors and muscarinic receptors decreased [72, 73], indicating that RasGRF1 downregulation was accompanied by a decline in neurotransmitter receptor activation, resulting in impaired LTP.

Structural plasticity of neurons is another key component of synaptic plasticity. RasGRF1 can participate in regulating neuronal structural plasticity. Benzothiazole aniline promotes dendritic spine formation through the RasGRF1-Ras dependent pathway [20]. Very low-density lipoprotein also promotes RasGRF1-mediated spinogenesis [19]. Mercaptoacetamide-based class II histone deacetylase inhibitor increases dendritic spine density via the RasGRF1/ERK pathway [21]. These findings suggest that RasGRF1 can regulate the structural plasticity of dendritic spines. In addition, BDNF promotes axonal growth, and neurotrophin facilitates neurite outgrowth through RasGRF1 activation [18, 22, 74]. Moreover, RasGRF1 regulates dendritic branching in hippocampal neurons [75]. Taken together, all of these findings suggest that RasGRF1 plays an important regulatory role in neuronal structural plasticity. The present study showed that *Rasgrf1* downregulation was accompanied by a decrease in dendritic spine numbers and synaptic densities and the deterioration of synaptic structure after CCH, but these effects were reversed following *Rasgrf1* upregulation by miRNA-323-3p inhibition. Thus, this indicates that RasGRF1 downregulation may contribute to the reduction in structural plasticity and subsequent cognitive impairment after CCH, and RasGRF1 upregulation could improve them. Furthermore, a previous study showed RasGRF1 could regulate neurogenesis because *Rasgrf1* knockout mice displayed severe neurogenesis and dendritic arborization [76]. As neurogenesis contributes to cognitive plasticity, RasGRF1 downregulation may impair cognition after CCH by inhibiting neurogenesis. This should be investigated in future research.

## CONCLUSIONS

In conclusion, the present study showed that *Rasgrf1* was regulated by miRNA-323-5p and that RasGRF1 downregulation may contribute to spatial learning and memory impairment after CCH. Meanwhile, *Rasgrf1* upregulation via miRNA-323-5p inhibition may ameliorate cognitive impairment by promoting synaptic plasticity, providing a potentially valuable and effective therapeutic target for cognitive dysfunction after CCH.
